# Design and *in Vitro* Biocompatibility of a Novel Ocular Drug Delivery Device

**DOI:** 10.3390/jfb4010014

**Published:** 2013-01-18

**Authors:** Nathan Gooch, Randon Michael Burr, Dolly J. Holt, Bruce Gale, Balamurali Ambati

**Affiliations:** 1 Department of Bioengineering, University of Utah, Salt Lake City, UT 84112, USA; 2 Moran Eye Center, University of Utah, Salt Lake City, UT 84132, USA; E-Mails: rmikeburr@gmail.com (R.M.B.); bala.ambati@utah.edu (B.A.); 3 Department of Cardiothoracic Surgery, University of Utah, Salt Lake City, UT 84112, USA; E-Mail: Dolly.Holt@utah.edu; 4 Department of Mechanical Engineering, University of Utah, Salt Lake City, UT 84112, USA; E-Mail: bruce.gale@utah.edu

**Keywords:** CDR, capsular bag, drug delivery, biocompatibility

## Abstract

The capsule drug ring (CDR) is a reservoir and delivery agent, which is designed to be placed within the capsular bag during cataract surgery. Prototypes were manufactured by hot melt extrusion of Bionate II^®^, a polycarbonate urethane. The devices have been optimized using Avastin^®^ as the drug of interest. *In vitro* biocompatibility was assessed with human lens epithelial cell (B-3), mouse macrophage (J774A.1) and mouse fibroblast (L-929) cell lines. Cell migration and proliferation were assessed after *in vitro* culture. Pro-inflammatory cytokines (*i.e.*, MIP-1β, MIP-1α, MCP-1, IL-1β, TNF and TGF-β1) were quantified using cytometric bead array (CBA). Preliminary *in vivo* biocompatibility and pharmacokinetics testing has been performed in rabbits.

## 1. Introduction

Of the diseases that result in vision loss, those which affect the retina and retinal function are of particular research interest due to the resultant permanent loss of visual function for which there is no definitive treatment. Current therapies restrict the progression of the disease, but due to limitations inherent in current pharmaceutical delivery modalities, cannot cure it [1,2,3,4,5]. Continuous or repeated treatment is an implication of this form of therapy. Age-related macular degeneration (AMD) is the leading cause of blindness and significant visual impairment in developed nations. There are 30 million worldwide cases of AMD; in the United States, there are over 2 million current cases of advanced AMD, with 200,000 new cases every year [3]. AMD is expressed in two principle forms. The “wet” or exudative form of the disease is characterized by the formation of new blood vessels, also known as angiogenesis. The “dry” or non-exudative form of the disease is characterized by the formation of drusen and often progresses into wet AMD. Rates of blindness due to retinal degeneration are expected to rise as the population ages over the next few decades [1].

**Figure 1:**
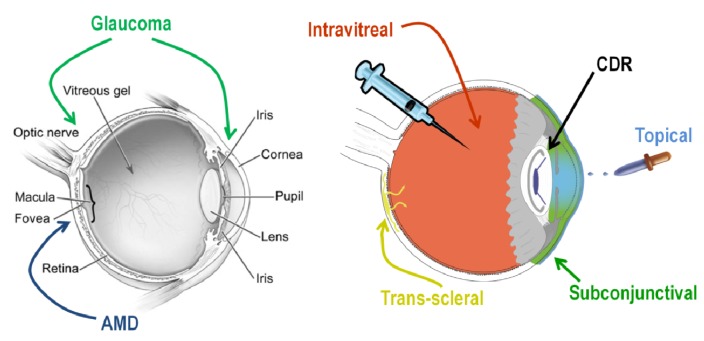
Age-related macular degeneration (AMD) and Glaucoma methods of treatment are shown.

The primary location of pharmaceutical action for the treatment of AMD is the posterior segment and the retina, with particular focus on the retinal pigment epithelium (RPE). The primary goal of treatment is to preserve the macula. While there are multiple methods of treatment under investigation, the current clinical treatment for this disease is indefinite frequent intravitreal injections (Figure 1).

Current treatments of AMD focus on pharmacological approaches. Due to the impact of angiogenesis in wet AMD, a significant amount of successful research has been focused on the use of anti-vascular endothelial growth factor (anti-VEGF) therapies as viable treatments for patients [2,3,4,5,6,7,8]. However, the current method of pharmaceutical delivery is indefinite intravitreal injections, which are performed as often as monthly, which can result in resistance [3,9]. These injections must be performed by retinal specialists due to the serious risks associated with these injections (e.g., retinal detachment, endophthalmitis and vitreal hemorrhaging). Modern anti-angiogenic therapies offer significant benefit to many patients with neovascular AMD, but would be much improved with a drug delivery modality that supports sustained and extended release profiles. Improvement in the treatment of AMD and other ocular diseases can have an immediate positive impact on the quality of life of patients. 

Currently, there are implantable intraocular drug delivery devices on the market, but none of these commercialized ocular drug delivery devices deliver anti-VEGF; nor are they refillable, versatile and implantable by general ophthalmologists. In addition, these devices target AMD, which is the leading cause of blindness in the United States [10,11,12,13,14,15,16,17,18]. The Capsule Drug Ring (CDR) is a novel drug delivery device, which is focused on the treatment of wet AMD by the sustained delivery of anti-VEGF. The device is designed to reside within the unused periphery of the capsular bag after cataract surgery. This can be implanted during standard intraocular lens (IOL) implantation, which eliminates the need of an additional surgery. The CDR resides in the anterior chamber’s capsule bag, which reduces implantation and refilling costs, as retinal specialists are not needed. This device is intended to be a permanent, refillable implantation. This approach takes advantage of the frequency of cataract surgeries (3,000,000 annually in the United States), eliminating the need for additional surgeries or sutures. The incorporation of long-term release kinetics will be a driving factor in the success of this device. Lucentis costs approximately $2,500 per injection (prospective annual cost approximating $30,000 per patient); Avastin costs approximately $150 per injection (prospective annual cost approximating $1,800 per patient). The CDR has been designed with the goal of reducing the frequency of injections, thereby reducing the cost of treatment. With the goal of reducing the number of injections from monthly to once every six months, the annual cost of AMD treatment could be reduced by as much as 50%.

To test our idea of an implantable permanent intraocular drug delivery device we chose materials, which have shown *in vivo* biocompatibility in other bodily tissues, and screened their *in vitro* ocular biocompatibility via cell culture. We also tested the ability of Avastin, a ~150 kDa antibody, to permeate from the anterior chamber to the posterior chamber of the eye. Finally, we studied preliminary *in vitro* and *in vivo* drug release kinetics from the CDR.

## 2. Experimental Procedures

### 2.1. Materials and Design

**Figure 2:**
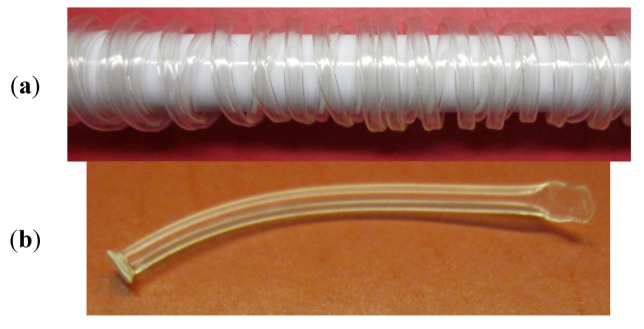
(**a**) Bionate II the tubing after hot melt extrusion showing curvature; (**b**) a linear section showing the attached membrane and the seal of the opposing end.

The CDR devices were manufactured of three materials: Bionate II^®^, polyethersulfone and Loctite^®^ 4307™. Bionate II (DSM-Biomedical, Berkeley, CA, USA) 80A, a polycarbonate urethane (PCU) was synthesized by DSM Biomedical into pellets using DSM’s proprietary technology and subsequently extruded into tubes using hot melt extrusion (Haake Minilab II Micro Compounder, Thermo Scientific, Waltham, MA, USA). Tubing was extruded using a long land die (Guill Tool and Engineering, West Warwick, RI, USA) with a 0.750 outer diameter (420 SS) and a bullet nose tip. As the Bionate II tubing was extruded from the dye, it was wrapped around an 8 mm pipe to incorporate the correct inner and outer diameters into the polymer before fully setting. This extrusion/die setup formed tubing with an outer diameter of 1.4 mm, a wall thickness of 0.2 mm and an inner diameter of 1.0 mm (Figure 2). Additional Bionate II films were extruded to a thickness of approximately 250 μm for use in biocompatibility assays. All the materials were synthesized and extruded without extrusion additives. Bionate II tubing was coated with Vitrostealth^®^ (DSM-Biomedical, Berkeley, CA, USA), a polyethylene glycol coating, after extrusion using DSM’s proprietary technology.

Polyethersulfone (PES) (Sterlitech, PES0032005) was purchased from Sterlitech Corporation (Kent, WA, USA). The membrane has passed USP Class VI tests and is commonly used for hemodialysis membrane filters. The PES filter had 30nm pore-sized holes at a density of ~70%. Loctite 4307 (Henkel Corporation, Düsseldorf, Germany), a UV sensitive medical grade adhesive, has a history of being used in medical devices and is ISO 10993 compliant. This material served as the medium of adhesion between the Bionate II tubing and the PES membrane. The CDR’s were assembled by cutting the Bionate II tubing into 360° segments, yielding a circular area of 0.79 mm^2^. PES membranes were cut to fit around one of the two open ends of the tubing segment and adhered into place using Loctite 4307. The other end of tubing was sealed closed using a heat press. CDRs were then cleansed by rinsing the devices three times in 200 proof ethanol. The devices were dried between each rinse. The devices were then sterilized using standard ethylene oxide treatment methods as part of a larger batch for the University of Utah medical hospital.

### 2.2. Endotoxin Assessment

After manufacture, the devices were tested for presence of endotoxin. CDR device components and fully manufactured devices were placed in LAL water in separate endotoxin free containers and placed on a shaker plate at room temperature for 3 days. The samples were then placed in sonication for two hours and thirty minutes at 37 degrees Celsius. Meanwhile, a standard curve was formed from known endotoxin concentrations of 0, 0.005, 0.1, 0.25, 0.5, 1, 5 and 50 EU/mL. Afterwards, the rinsate from the samples was extracted. The filter began to degrade after sonication and was centrifuged at 13,000 RCF to remove debris, and the supernatant was used for the experiment. Each sample rinsate was read using a microplate reader at 405 nm. The reaction onset time of each sample was recorded at n = 3. The onset time is the time taken to reach the OD value (usually 0.03 OD units). Using the standard curve, the endotoxin levels of the samples were determined.

### 2.3. *In vitro* Cytotoxicity

*In vitro* cytotoxicity of the device components was assessed by the incubation (37.0 °C; air, 95%; carbon dioxide (CO_2_, 5%) of J774A.1 mouse macrophage and L-929 mouse fibroblast cell lines onto the major components of the CDR’s. J774A.1 macrophages and L-929 fibroblasts and appropriate growth media were purchased from ATCC. J774A.1 macrophages were incubated in 10% fetal bovine serum in Dulbecco’s Modified Eagle’s Medium. L-929 fibroblasts were incubated in 10% horse serum in Eagle's Minimum Essential Medium. CDR components were sterilized using standard ethylene oxide treatment methods, as previously discussed prior to cell plating. Cell media was harvested each day for 5 days at which time negative controls had reached full confluency. Inflammatory cytokines (*i.e.*, MIP-1β, MIP-1α, MCP-1, IL-1β, TNF and TGF-β1) secreted by cells into media were quantified using cytometric bead array (CBA) as a measure of the *in vitro* biocompatibility of these materials. Each *in vitro* cytotoxicity assessment was performed in the absence of Avastin. While it is possible that the presence of this anti-VEGF pharmaceutical agent could impact the biocompatibility of the CDR device, eliminating the use of Avastin in this study allows for a clearer picture of the impact of each device material. *In vitro* cytotoxicity was evaluated in the context of surface roughness and surface energy. Surface roughness was determined using a Tencor P-10 Surface Profilometer. Surface energy was inferred through the measurement of interfacial contact angles of deionized water on each surface using an AmScope MD900.

### 2.4. Drug Release Kinetics—*In vitro*

To predict the *in vivo* release kinetics of Avastin from the CDR, several *in vitro* release studies were conducted ranging from 1 to 4 months with novel Avastin formulations. Avastin was frozen at −80 °C and lyophilized overnight. High molecular weight polyvinyl alcohol (140–158 kDa, Sigma, St. Louis, MO, USA) was added to balanced salt solution (BSS), a commonly used irrigating solution isotonic to the eye, at a final concentration of 50 mg/mL. The BSS was heated to 85 °C and stirred vigorously as the PVA was slowly added until completely solubilized. The PVA solution was cooled to room temperature, and the Avastin lyophilate was added to a final concentration of 100 mg/mL. Approximately 35 μL of the formulations were filled in Bionate extruded tubes with polyethersulfone (PES) membranes attached to one end by UV curing adhesive and the other end sealed by heat press after filling (Figure 2). Each tube was immersed in 4 mL of BSS in closed vials and stored at room temperature on an oscillatory shaker at low speed. Samples from each vial were taken at predetermined time points, and vial volume was maintained by addition of fresh BSS, thus maintaining sink conditions. A sample size of 3 was used per formulation tested.

### 2.5. Drug Release Kinetics—*In vitro*

A preliminary *in vivo* drug distribution study was conducted following the *in vitro* studies to verify if therapeutically relevant quantities of Avastin can be achieved in the retina/choroid. CDRs were sterilized using ethylene oxide and filled with drug the night before surgery. Standard cataract surgery with phacoemulsification was performed on all rabbits. CDRs were filled with ~35 µL of the aforementioned reformulated Avastin. Of the 8 rabbits used, 2 were sacrificed at the time points of 1, 4, 8 and 12 weeks. Upon sacrifice, tissues were segmented into the iris, cornea, sclera, retina, choroid, aqueous (anterior) and vitreous (posterior) humor and stored separately at −80° C.

Ocular drug distribution was gathered upon sacrifice of the rabbits at the predetermined time points. These data can be seen in Figure 7. Histopathology was collected and showed some signs of long-term inflammation and retinal hemorrhaging (data not shown). As a small preliminary study, it is unclear if the tissue aberrations are a result of the phacoemulsification/cataract surgery, CDR material/surgery or some other factor.

Both *in vitro* and *in vivo* Avastin sample concentrations were measured using an enzyme-linked immunoabsorbant assay (ELISA). Avastin concentration was detected with a goat antihuman IgG/Fc antibody labeled with horseradish peroxidase (Pierce Biotechnology Inc., Rockford, IL, USA) and chemiluminescent signal was detected on the EL800 Absorbance Microplate Reader (Biotek, Winooski, VT, USA).

## 3. Results and Discussion

Despite not being currently FDA approved for ocular applications, Avastin (bevacizumab; Genentech, San Francisco, CA, USA) was used as the pharmacological agent in this study. Lucentis® (ranibizumab; Genentech, San Francisco, CA, USA), the Fab fragment of Avastin, is an FDA-approved treatment for AMD. While Lucentis is a prescription medicine for the treatment of AMD, Avastin is being investigated as a lower-cost potential alternative therapy and is often used off-label for the clinical treatment of AMD [3]. Recent studies show similar outcomes in the ocular anti-angiogenic efficacies and safety of the two drugs [3].

### 3.1. Manufacture

**Figure 3:**
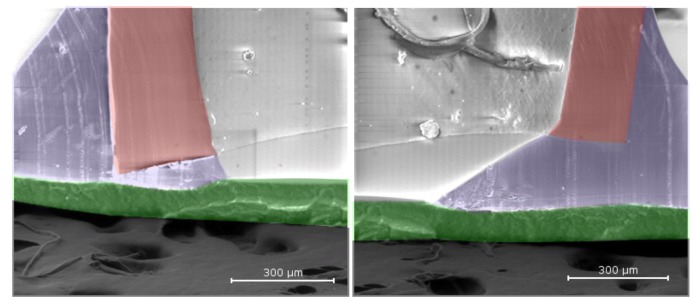
Standard electron microscopy (SEM) images show appropriate adhesion of Bionate II tubing using to the device membrane. Bionate II is shown in red, UV adhesive is shown in blue, the polyethersulfone (PES) membrane is highlighted in green and the SEM mount is shown in dark grey.

Our CDR devices were designed and manufactured to be placed within the capsular bags of cataract patients prior to IOL insertion. In order to be successful, the devices have very specific size requirements. To that end, polymer tubes were extruded with an outer diameter of 1.4 mm, a wall thickness of 0.2 mm and an inner diameter of 1.0 mm. This allowed for the devices to fit around the periphery of the capsular bag, maximizing reservoir size without obstructing vision. Standard electron microscopy (SEM) was performed on the devices to show appropriate adhesion of Bionate II to PES filters to confirm that the reservoir contents were not leaking from the device, but were indeed being released only through the membrane, which was carefully selected to generate our desired drug release kinetics. SEM also confirmed that adhesive did not foul our PES filter restricting drug flow from the device (Figure 3).

### 3.2. Endotoxin Assessment

**Figure 4:**
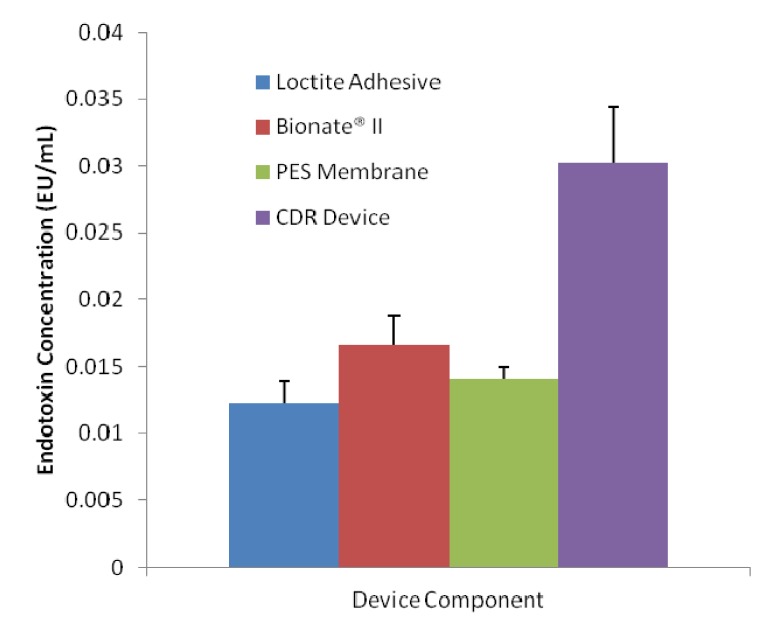
Endotoxin contamination levels of each component of the Capsule Drug Ring (CDR). Each component is at levels that are considered to be endotoxin free (< 0.5 EU/mL).

Manufactured CDRs should be free of adventitious microbial agents for the protection of ocular tissues and for the reduction of the host inflammatory and immune responses. Adventitious microbial could be introduced by the raw CDR materials or the manufacture process. In an attempt to reduce microbial presence, the CDRs were treated with ethanol washes and ethylene oxide after manufacture. In order to determine the efficacy of this method of cleansing, the manufacture method of the device and the raw CDR materials were tested for contamination with endotoxin. Endotoxins are the lipo-polysaccharides (LPS) from Gram-negative bacteria. Endotoxin assays test for the presence of viable Gram-negative bacteria and additionally detect the LPS from dead Gram-negative bacteria. LPS is one of the most common causes of toxic reactions due to the presence of pyrogens. Therefore, the absence of detected LPS indicates an absence of pyrogens. For this study, samples of each material and fully manufactured CDRs were quantified for endotoxin presence using a Limulus Amebocyte Lysate (LAL) assay. The result of the assay is shown in Figure 4. Each sample contained very low levels of endotoxin. The highest concentration of detected endotoxin in our samples was 0.0344 EU/mL, which is a full order of magnitude lower than what is considered to be endotoxin free, 0.5 EU/mL. The assay also detected less than half of the endotoxin contamination in each device component when compared to the fully manufactured devices. This would indicate that the method of device manufacture is introducing some small degree of bacterial contamination, but contamination levels are still well below what is considered endotoxin free.

### 3.3. *In vitro* Cytotoxicity

The interface between an implanted material and the surrounding biological tissue is the site of action wherein the host’s response to an implant is most markedly manifested. Materials implanted into both hard and soft tissues generate some degree of cellular response. The severity of the host’s response to an implant can be attributed to a number of factors, including the surgical technique, the size, shape and surface properties of the implant and the nature of the tissues at the implant location. The interfacial interactions are a major factor in determining the success or failure of an implant. Therefore, the determination of an implant’s impact on the host tissues is important to evaluate. This impact can only fully be anticipated through statistically powered *in vivo* usage of the implant; however, first step biocompatibility testing of biomedical devices can be shown through the use of *in vitro* cytotoxicity testing, as discussed in ISO 10993. Implantation into a host tissue generates a host cellular response. Activated cells produce cytokines, including MCP-1, TGF-β1, IL-1β, TNF, MIP-1α and MIP-1β. These cytokines are influential in regulating the host’s wound healing response. Wound healing involves a number of cells, including fibroblasts, monocytes, macrophages and endothelial cells. For this study, we performed cell culture with L-929 fibroblasts and J774A.1 macrophages. Both cell lines were individually cultured on the primary component of the CDR, Bionate II (DSM). Cell culture media was harvested after each day of the experiment, and assay cytokines were quantified through the use of CBA on media harvested from days 1, 3 and 5. Fibroblast cell culture media was quantified for the presence of MCP-1 and TGF-β1; macrophage cell culture media was quantified for the presence of MCP-1, TNF, MIP-1α and MIP-1β cytokines. These cytokine concentrations were compared to concentrations produced by cells cultured on incubation gold standard tissue culture polystyrene (TCPS). 

**Figure 5:**
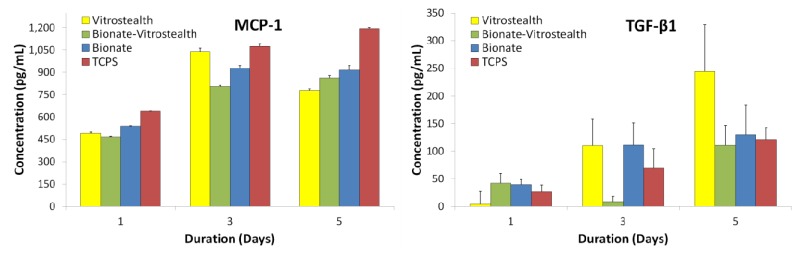
MCP-1 and TGF-β1 proinflammatory cytokines produced by L-929 fibroblasts were quantified by cytometric bead array (CBA) and are compared to cells cultured on tissue culture polystyrene.

L-929 fibroblasts were incubated on each surface and growth media was quantified 1, 3 and 5 days post-incubation for inflammatory cytokines (Figure 5). The production of MCP-1 and TGF-β1 proinflammatory cytokines had a general increasing trend over incubation time, which was to be expected as the cell populations increased over time. It was expected that a PEG coating (Vitrostealth) of the biomaterial would decrease the production of proinflammatory cytokines, but this correlation was not seen with our data. The production of TGF-β1 appeared to increase most dramatically for the fibroblasts cultured with the Vitrostealth coating. For both fibroblast produced quantified cytokines, the cellular populations appear to show little difference from the negative control.

**Figure 6:**
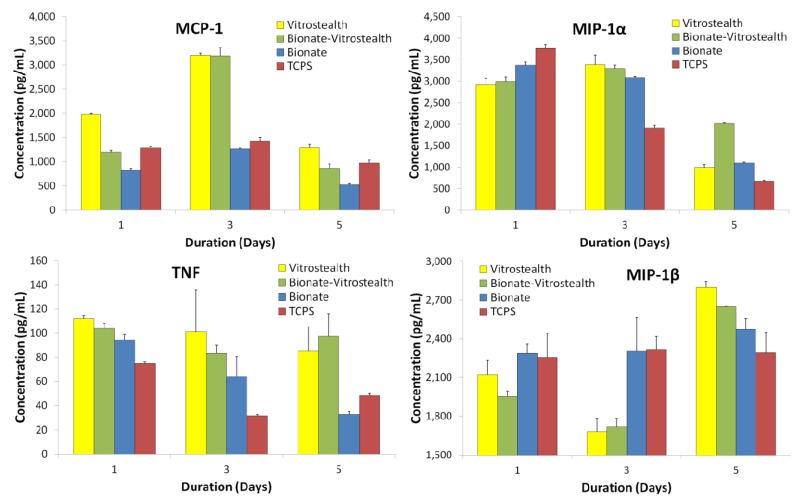
MCP-1, TNF, MIP-1α and MIP-1β proinflammatory cytokines produced by J774A.1 macrophages were quantified by CBA and are compared to cells cultured on tissue culture polystyrene.

J774A.1 macrophages were cultured experimentally similar to the L-929 fibroblasts, and media was harvested and quantified for days 1, 3 and 5. MCP-1, TNF, MIP-1α and MIP-1β proinflammatory cytokines were measured using CBA (Figure 6). Cells cultured on the Bionate II material tended to show similar cytokine concentrations to the negative control throughout the duration of the experiment. In addition, the macrophage cells cultured on each surface did not show signs of proliferative or morphological toxicity. This would indicate that the polycarbonate urethane (Bionate II) was not aggravating the cells. However, cells that were cultured on Vitrostealth generally tended to express higher concentrations of TNF and MCP-1. However, this increase in TNF (~100 pg/mL) and MCP-1 (~60 pg/mL) is not significant on these volume scales. While no material is perfectly biocompatible, these *in vitro* data indicate that each of the tested biomaterials has a very similar impact on cultured fibroblasts and macrophages as the gold standard in cell culture, TCPS. This is seen in the similar levels of cytokine production in each of the samples.

It should be noted that cellular adhesion is also an important indicator of material biocompatibility. During this study, this property was not implicitly measured for quantitative analysis, but a qualitative analysis shows cellular adhesion on each material surface to be comparable. Macrophage cell adhesion was unaffected by the material type, showing similar growth and adhesion on each of the materials. Fibroblast cellular adhesion appears to be impacted by each of the materials, resulting in a slight morphological change despite remaining viable. 

Surface roughness was measured for samples of TCPS, Bionate II and Vitrostealth-coated Bionate II. Contact angle measurements were also taken for TCPS and Bionate II samples. These data are shown in Table 1. The data indicate that Bionate II was relatively rough in comparison to the other two surfaces. This may have induced some degree of increased cellular toxicity in comparison to the other samples; however, surface contact angles for both Bionate II and TCPS were very similar, and the cellular release of proinflammatory cytokines also indicates that the increased roughness of the Bionate II samples had little effect on the cytotoxicity of the samples.

**Table 1:** Surface measurements of roughness and hydrophilicity.

Material	Surface roughness (Ra, Å)	RMS roughness (Rq, Å)	Contact angle (degrees)
TCPS	177.7 ± 46.6	227.1 ± 62.6	76.5 ± 2.3
Bionate II	754.3 ± 372.2	953.5 ± 452.3	77.0 ± 2.5
Vitrostealth	98.8 ± 36.4	123.1 ± 47.0	N/A

### 3.4. Drug Release Kinetics—*In vitro/in vivo*

The current standard of care for the treatment of AMD is monthly intravitreal injections of Lucentis (ranibizumab; Genentech). However, Avastin (bevacizumab; Genentech) is a drug that is increasingly being used off label as a replacement for Lucentis for AMD treatment. For our drug release kinetics assessments, we have used Avastin. For clinical treatment, each monthly bolus injection contains 1.25 mg, and thus, the daily rate delivered is 41.7 μg/day. We used this as an initial target rate to be further refined with future *in vivo* experimentation. It is unclear if more or less drug than this is needed, as the implant location is intended for the capsular bag as compared to intravitreally placed. Furthermore, sustained release drug delivery generally requires less drug, and therefore, a clinically relevant delivery rate will likely differ. Delineating macromolecular drug delivery from CDRs placed in the capsular bag at the time of cataract surgery is ongoing.

**Figure 7:**
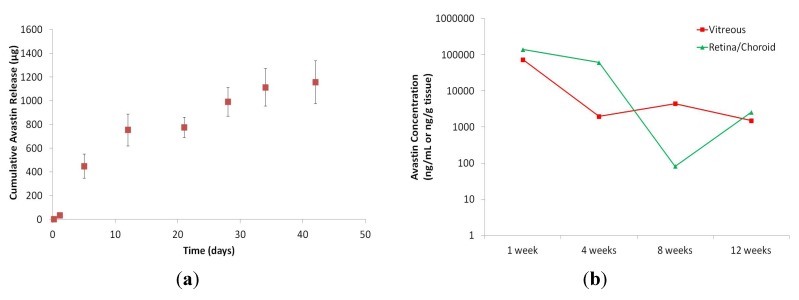
CDR sustained drug release was quantified by measuring Avastin release over time. (**a**) This figure shows *in vitro* release of Avastin over 40+ days; (**b**) *In vivo* release of Avastin was detected in rabbit tissues out to at least 12 weeks.

Drug release for the CDR was assessed *in vitro* and for a preliminary *in vivo* study. These experiments were performed as a first assessment of CDR device efficacy. *In vitro* media was harvested over time, as previously described, and assessed for Avastin concentrations at predetermined time points. The cumulative *in vitro* drug release accounts for ~30% of the total loaded drug, where release plateaued at day 42 (Figure 7). Explanations for the unaccounted drug include non-specific binding with the CDR, protein degradation from elevated temperatures and aggregation from the elevated concentration of 100 mg/mL. The presence of these phenomena were confirmed, but not quantified explicitly. This study showed a two phase drug release profile. The first 10 days show a drug release of about 80 μg/day. After the first 10 days, the rate of drug release slows down to about 16.5 μg/day. Future work with the CDR will include the tuning of the rate of drug release to incorporate near-zero order release kinetics and stability optimization, as measured by charge variance analysis, to maintain a therapeutic effect, as determined in an appropriate disease model.

*In vivo* drug concentrations were quantified after implantation into 4 rabbits, concurrent to our *in vitro * assessment. The rabbits were sacrificed at 1, 4, 8 and 12 weeks. Tissues were harvested and ELISA was performed. Ocular drug distribution was assessed upon sacrifice of implanted rabbits at predetermined time points, as shown in Figure 7. To the best of our knowledge, there is no published work showing the penetration of a large molecule from the anterior chamber to the posterior chamber. This experiment clearly shows the ability of a large molecule, such as Avastin (149 kDa), to penetrate into retinal tissues. During week 1, the drug concentration is found to be about 100 μg/mL and, over the course of 12 weeks, decreased to a concentration of about 1 μg/mL. However, for this experiment, only a single rabbit (n = 2 eyes) was assessed for each timepoint, and a statistically powered experiment will need to be performed in order to determine a correlation between the *in vitro* and *in vivo* release profiles.

## 4. Conclusions

Today AMD continues to be the leading cause of blindness and significant visual impairment in developed nations. While current treatments are able to slow the progression of the disease and improve the quality of life of many patients, the treatment process is far from perfect. Significant improvement can be made in patient health outcomes through the development of an extended release device. The development of the Capsule Drug Ring continues to be a work in progress, but preliminary results of key *in vitro* device biocompatibility and efficacy assessments demonstrate the potential of the device. Future work with the device includes the incorporation of Avastin into *in vitro* biocompatibility assays, development of improved drug release kinetics to improve the long-term efficacy of the device and the complete statistically powered *in vivo* biocompatibility and efficacy assessment of the device.

The results of this study show the successful manufacture of the CDR, a potentially refillable drug delivery device. The device is able to deliver Avastin out to 90+ days, while showing acceptable biocompatibility. With a strategy of refilling the device only once over this 90 day period, the burden of the cost of healthcare resulting from AMD treatments could be reduced by 25%. *In vitro* results show the devices and their individual components to be highly biocompatible with cells, showing little difference in migration, proliferation and pro-inflammatory cytokine generating behaviors when compared to gold standard culture methods. The CDR shows great potential as an implantable ocular device for drug delivery.
